# Coronary magnetic resonance angiography of coronary artery total occlusion: imaging findings and success rates of percutaneous coronary intervention according to intraluminal signal intensity patterns

**DOI:** 10.1186/1532-429X-16-S1-O56

**Published:** 2014-01-16

**Authors:** Sung Mok Kim, Yeon Hyeon Choe, Jin-Ho Choi

**Affiliations:** 1 Radiology, Samsung Medical Center, Seoul, Korea, Republic of; 2 Cardiology, Samsung Medical Center, Seoul, Korea, Republic of

## Background

We hypothesized that intraluminal signal intensity (SI) of coronary total occlusion (CTO) lesions at coronary MR angiography (CMRA) may reflect the degree of the softness of the lesion with or without the presence of microvessels. The purposes of our study were to report the CMRA findings of CTO lesions and to compare success rates of percutaneous coronary intervention (PCI) for CTO lesions according to different SI patterns at CMRA.

## Methods

Ninety-one consecutive CTO patients who underwent steady-state free precession CMRA (free-breathing whole-heart and breath-hold volume-targeted) and late gadolinium enhancement imaging using a 1.5-T scanner before PCI were included. We analyzed the images of CMRA and PCI results of the patients. Ninety-three CTO lesions were analyzed for this study. Lesions were graded according to CMRA findings of CTO segment: (Gr. 1) tubular appearance with homogeneously high SI (HSI), (Gr. 2) continuous HSI areas with moderate irregularity in contour, (Gr. 3) heterogeneous SI lesion with interrupted HSI areas, (Gr. 4) intermediate or low SI lesion. Lesions were classified into two groups with regard to the presence of the continuity of HSI areas. Angiographic findings including lesion length, collateral grades, and thrombolysis in myocardial infarction (TIMI) grades were recorded at invasive coronary angiography. Multivariable statistical test was performed to identify variables associated with successful PCI.

## Results

Seventy-three lesions (78%) of 93 CTO lesions were successfully treated with PCI. On CMRA images, the presence of continuity of HSI areas was found in 60 lesions (64.5%) including lesions of Gr. 2 (n = 13). Fifty-four lesions (90%) of the 60 lesions were successfully treated with PCI. The others (Gr. 3 or 4) were 33 lesions (Gr. 3, 14; Gr. 4, 19). Nineteen lesions (58%) of the 33 lesions were successfully treated with PCI. At multivariable analysis, the presence of continuity of HSI areas in CTO segments proved to be the only independent predictor of PCI success (odds ratio, 6.65: 95% confidence interval, [2.09, 21.11]; P = 0.001), while angiographic findings did not significantly correlate with PCI outcomes.

## Conclusions

The CMRA findings showing continuity of HSI areas in CTO segments predict better success rates of PCI.

## Funding

No.

